# Periodontitis and the risk of oral cancer: a meta-analysis of case-control studies

**DOI:** 10.2340/aos.v83.40478

**Published:** 2024-05-14

**Authors:** Yan Ma, Nijiati Tuerxun, Gulibaha Maimaitili

**Affiliations:** Department of Stomatology Xinjiang Medical University, Affiliated Hospital 2, Urumqi, Xinjiang 830063, China

**Keywords:** Periodontal diseases, periodontitis, oral cancer, missing teeth, clinical attachment loss, meta-analysis

## Abstract

**Objective:**

The current studies have yielded inconclusive findings regarding the connection between periodontitis and oral cancer (OC). Therefore, our goal is to elucidate this relationship.

**Materials and methods:**

We conducted a thorough search of electronic databases (EMBASE, PubMed, Web of Science, and Cochrane Library) up to September 2023. The Newcastle-Ottawa Scale (NOS) was applied to assess study quality. To evaluate potential publication bias, both a funnel plot and Egger’s test were employed. Additionally, a sensitivity analysis was conducted to explore the source of heterogeneity when the *I*^2^ statistic exceeded 50%.

**Results:**

This systematic review encompassed 16 studies, involving a total of 6,032 OC patients and 7,432 healthy controls. Our meta-analysis, incorporating data from nine studies, revealed a significant correlation between periodontitis and the risk of OC (OR [odds ratio] = 2.94, 95% CI [confidence interval] (2.13, 4.07); five studies, 6,927 participants; low certainty of evidence). Findings also suggested that individuals with more than 15 missing teeth may have a heightened risk of OC (OR = 1.91, 95% CI (1.01, 3.62)). Furthermore, clinical attachment loss (CAL) and decayed, missing, and filled teeth (DMFT) in OC patients were more pronounced compared to the control group (CAL, SMD = 1.94, 95% CI (0.22, 3.66); DMFT, SMD = 0.65, 95% CI (0.12, 1.18)).

**Conclusion:**

Periodontitis may serve as a potential risk factor for OC. However, caution is warranted in interpreting these findings due to the substantial level of heterogeneity.

## Introduction

Periodontitis is a progressive disease that develops over time due to the accumulation of biofilm at the gumline, coupled with the host’s compromised ability to effectively eliminate potentially harmful microflora [1]. Consequently, this process can lead to the sub-gingival colonisation of microbes, persistent inflammation, and damage to adjacent tissues, resulting in the formation of periodontal pockets attributed to the loss of connective tissue attachment and concurrent bone loss [2, 3]. Furthermore, substantial evidence links periodontitis to various systemic conditions, such as diabetes mellitus [4], cardiovascular diseases [5], poor pregnancy outcomes [6], and pulmonary, renal, and immunologic disorders [3]. Periodontal pathogens, such as Fusobacterium nucleatum and Porphyromonas gingivalis, are frequently identified in malignant growths within the oral cavity [7, 8], as well as other types of cancer [9]. These connections have been a subject of investigation for many years, with a recent surge in publications highlighting the connection between periodontitis and cancer risk.

Cancer remains a significant contributor to global mortality, with oral cancer (OC) ranking as the sixth most prevalent form worldwide [10]. The estimated global annual incidence of OC is approximately 500,000 cases, with over 60% of these reported in Asia and Pacific regions, including nations like India, Pakistan, Sri Lanka, Taiwan, and Papua New Guinea [11]. Remarkably, OC constitutes approximately 25% of cancer treatment cases in these high-risk countries [10]. Recognised risk factors for OC include well-established factors such as tobacco and alcohol use [12], as well as systemic conditions like diabetes mellitus [13], metabolic syndrome [14], and chronic inflammation and infection [15]. Moreover, numerous narrative reviews have discussed the links between tooth loss, periodontal disease (PD), and cancer [16–18]. Research has indicated that individuals with PD face an increased risk of developing various cancer types, including pancreatic [19], skin [20], colon [21], and breast [22], compared to those without PD. Dental biofilms and the accompanying dysbiosis are implicated in several oral conditions, including OC [23, 24]. Two meta-analyses evaluating the relationship between PD and OC, with limited numbers of the included studies (ranging from 5 to 11 studies) [25, 26] demonstrated this positive association. A recently published meta-analysis has additionally shown that tooth loss may nearly double the risk of OC [27].

Therefore, with the emergence of recent publications, the objective of this study is to conduct a comprehensive meta-analysis of all pertinent published literature, seeking to explore the potential correlation between PD (specifically, periodontitis) and OC.

## Materials and methods

This study adhered to the Preferred Reporting Items for Systematic Reviews and Meta-Analyses (PRISMA) guidelines, involving a systematic process that encompassed a thorough literature search, meticulous organisation of review documents, rigorous assessment of the quality of each empirical study, comprehensive data synthesis, and meticulous report writing. Additionally, our meta-analysis was registered in the International Prospective Register of Systematic Reviews (PROSPERO) with the registration number CRD42023407582.

### Literature search

We systematically conducted a literature search across multiple databases, including PubMed (National Library of Medicine, Washington, DC), Embase, Cochrane, and Web of Science, to gather studies specifically investigating the association between periodontitis and OC. The search spanned from the inception of the databases up to September 2023, utilising a strategy that combined Medical Subject Headings (MeSH) terms with their corresponding free-text terms. The MeSH terms employed in the search included ‘oral cancer’, ‘periodontal disease’, ‘tooth loss’, ‘squamous cell carcinoma’, ‘missing teeth’, ‘alveolar bone loss’, ‘clinical attachment loss’, and ‘periodontitis’. The complete search strategy is detailed in Table S1.

### Inclusion criteria and exclusion criteria

The selection criteria for this study were established in accordance with the PECO framework, which delineates the key components as follows: P (any population), E (exposed to a history of periodontitis or OC), C (comparator, not exposed to periodontitis or OC), and O (outcome indicators related to OC). In essence, our investigation focused on observational research, comprising both case-control and cohort studies, exploring the association between various indicators of periodontal health and OC. Eligible studies had to meet the following conditions: (1) Clearly defined control and case groups, with patients in the case group having a well-established diagnosis of periodontitis or OC, using specified tools such as the International Classification of Diseases, Ninth Revision, Clinical Modification (ICD-9-CM) [28] for periodontitis and the ICD for Oncology [29] for OC; and (2) inclusion of hazard ratios (HRs), odds ratios (ORs), or risk ratios (both unadjusted and adjusted) accompanied by 95% confidence intervals (CIs), or the provision of sufficient data within the article to facilitate the computation of these ratios.

The exclusion criteria were defined as follows: (1) Studies lacking explicit diagnostic and effectiveness criteria; (2) Studies categorised as meta-analyses, review articles, case reports, conference abstracts, guidelines, letters/response to the editors, or opinion articles; and (3) Studies containing incomplete or inaccurate data that precluded meaningful integration.

### Study selection and data extraction

Following the criteria for inclusion and exclusion outlined previously, the study selection process was independently conducted by two researchers, Yan Ma and Nijiati Tuerxun. Initially, all potentially relevant studies were imported into EndNote X9 to identify and eliminate duplicate entries. Subsequently, a screening process ensued, involving the review of titles and abstracts to exclude studies that did not meet the predefined eligibility criteria. Finally, full-text articles underwent additional screening. In cases of disagreements, resolution was attained through discussion or consultation with a third researcher, Gulibaha Maimaitili.

We utilised the Cochrane data extraction form to retrieve the following data and details: (1) Fundamental information including the title, primary author’s name, and publication year; (2) Essential characteristics of the study subjects, encompassing age, gender, case numbers in each group, and diagnostic criteria; and (3) Outcome measures, which encompassed the relationship between tooth loss, squamous cell carcinoma, alveolar bone loss, or clinical attachment loss (CAL), and OC.

### Quality assessment of the included studies

To evaluate potential bias and the overall quality of the included studies, we employed the Newcastle–Ottawa Scale (NOS), a widely utilised tool for assessing the methodological quality or risk of bias in case-control studies, in accordance with Cochrane recommendations [30, 31]. The evaluation of publication bias in the included studies was independently conducted by two researchers, Yan Ma and Nijiati Tuerxun, with any discrepancies resolved through consultation or discussion with a third researcher, Gulibaha Maimaitili.

Each included study underwent an evaluation based on specific criteria. For case-control studies, these criteria included selection (scored from 0 to 4), comparability (scored from 0 to 2), and exposure (scored from 0 to 3). For cohort studies, the criteria consisted of selection (scored from 0 to 4), comparability (scored from 0 to 2), and outcome (scored from 0 to 3). The results were then interpreted according to commonly accepted standards and categorised into the following groups: very high risk of bias (0–3 NOS points), high risk of bias (4–6 NOS points), and low risk of bias (7–9 NOS points) [32].

### Data analysis

We calculated the combined OR for OC in individuals with PD compared to those without PD, accompanied by the associated 95% CI, using Stata version 16.0. Simultaneously, we assessed the standardised mean difference, along with a 95% CI, to examine various forms of PD between OC patients and healthy controls. Heterogeneity was evaluated by quantifying the proportion of variation attributed to confounding variables, employing *I*^2^ statistics. An *I*^2^ value exceeding 50% indicated a significant degree of heterogeneity among the included studies. In such instances, a random-effects model was employed, and a sensitivity analysis was conducted to identify the source of this heterogeneity. Conversely, when *I*^2^ was below 50%, a fixed-effects model was applied. Additionally, suspicion of publication bias was raised if the *p*-value was below 0.05.

## Results

### Literature search results

**Figure 1:**
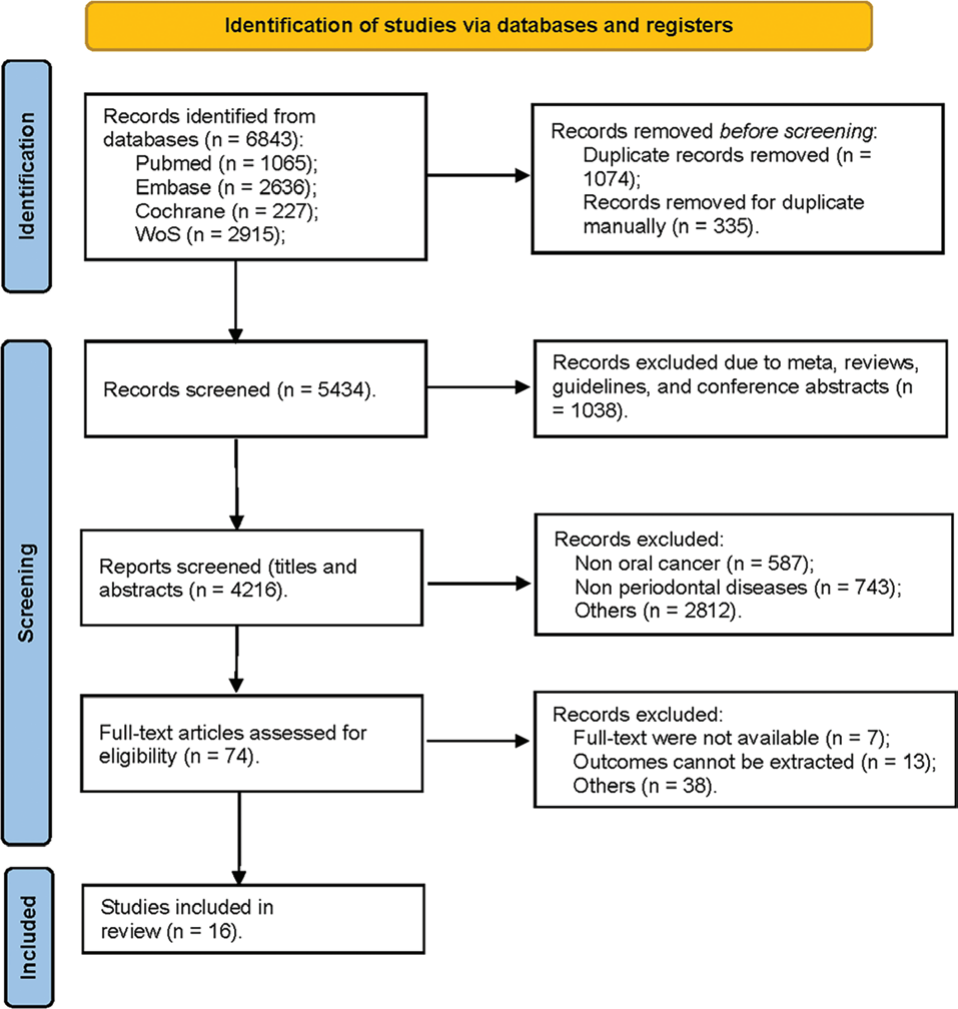
Flow diagram for searching and selecting qualifying studies incorporated in the meta-analysis.

Following the search strategy, a total of 6,843 records were initially identified from the four databases, with an additional four records recognised through relative reviews. After removing 1,409 duplicates, the remaining 5,434 records were screened, leading to the removal of 1,038 records categorised as meta-analyses, reviews, guidelines, or conference abstracts. Subsequently, a review of the titles and abstracts of the remaining records led to the removal of 4,142 records based on the predefined criteria for inclusion and exclusion mentioned earlier. Full-text articles were evaluated, and 58 records were excluded due to unavailability of full texts and relevant outcomes. Ultimately, the present study incorporated a total of 16 studies. The detailed selection process is displayed in Figure 1.

### Description of the included studies

Table 1 presents a comprehensive overview of the eligible studies, delineating key characteristics such as the country of origin, study design, sample size, case and control group sizes, types of cases, and outcome indicators. Specifically, the analysis encompasses 12,584 case-control studies on OC, including OC [33–37], oral squamous cell carcinoma (OSCC) [38–43], oral cavity and/or oropharyngeal cancer (OOSCC) [44–46], and pharyngeal cancer [47], and 19,273 healthy controls. Among the included studies, six were conducted in Asia region [35, 36, 39, 40, 47, 48], five in European regions [38, 41–43, 46], two in the United States of America (USA) [37, 45], and three in other countries [33, 44, 49]. Smoking as a known risk factor for both OC and PD has been adjusted in the included studies when evaluating the association between periodontitis and OC. Besides, other adjusted factors such as age, gender, and alcohol consumption have also been adjusted and are presented in Table 2.

**Table 1:** Main characteristics of the included studies in the meta-analysis.

Included studies	Country	Study design	Case	control	OC kinds	Evaluation of PD
Komlos et al. (2021)	Hungary	Case-control	100	100	OSCC	CAL, PPD, BOP, SLPI, DMFT
Chen et al. (2021)	China	Case-control	1292	2584	Oropharyngeal cancer	SRP, History of periodontitis
Shin et al. (2019)	Korea	Case-control	146	278	OSCC	Radiographic ABL
Laprise et al. (2016)	Canada	Case-control	350	371	OC	Gingival inflammation, Gingival recession
Moraes et al. (2016)	Brazil	Case-control	35	40	OC	Chronic periodontitis, DMFT, plaque index, Gingival index, PPD, CAL
Narayan et al. (2014)	India	Case-control	242	254	OSCC	Dental caries, oral hygiene status, and periodontal disease status.
Moergel et al. (2013)	Germany	Case-control	178	123	OSCC	MBL, missing teeth
Tezal et al. (2009)	USA	Case-control	267	207	OC	ABL, missing teeth
De Rezende et al. (2008)	Brazil	Case-control	50	50	OOSCC	Dental caries, missing teeth
Tezal et al. (2007)	USA	Case-control	51	54	OOSCC	ABL, missing teeth, decayed teeth, filled teeth, crowns, root canal treatment
Chang et al. (2007)	China	Case-control	317	296	OC	Regular dental visits, missing teeth
Guha et al. (2007)	France	Case-control	2286/924	1805/928	OSCC	Oral hygiene, missing teeth, Dental check-ups
Rosenquist et al. (2005)	Sweden	Case-control	132	320	OOSCC	Missing teeth
Garrote et al. (2001)	Cuba	Case-control	200	200	OC	Dental check-ups, missing teeth
Bundgaard et al. (1995)	Denmark	Case-control	161	483	OSCC	Dental check-ups, numbers of teeth
Zheng et al. (1990)	China	Case-control	248	248	OC	Missing teeth,

OC, oral cancer; PD, periodontitis; OSCC, oral squamous cell carcinoma; ABL, alveolar bone loss; CAL, clinical attachment loss; PPD, periodontal pocket depth; BOP, bleeding on probing; SLPI, Silness-Löe plaque index; DMFT, decayed, missing and filled teeth; SRP, Periodontitis and scaling and root planning.

**Table 2:** Adjusted factors when evaluating the association between periodontitis and OC in the included studies.

Included studies	Adjusted factors
Shin et al. (2019)	age, sex, and smoking, alcohol intake, education level, physical activity, obesity, diabetes, hypertension, and hypercholesterolemia.
Laprise et al. (2016)	paan chewing, bidi and cigarette smoking, alcohol consumption, age, gender and number of educational years
Moraes et al. (2016)	smoking and alcohol
Tezal et al. (2009)	age at diagnosis, gender, race/ethnicity, marital status, smoking status, alcohol use, ABL, and missing teeth.
Tezal et al. (2007)	smoking and alcohol
Rosenquist et al. (2005)	tobacco smoking and alcohol consumption
Chen et al. (2021)	
Chang et al. (2007)	sex, age, education, cigarette smoking (pack-year categories), betel quid chewing (pack-year categories), and alcohol drinking (frequency) sex, age, education, cigarette smoking (pack-year categories), betel quid chewing (pack-year categories), and alcohol drinking (frequency)

OC, oral cancer.

### The quality assessment of the included studies

The evaluation of bias risk revealed that, among the papers included in the analysis, the mean quality score based on NOS was 8.2 for the 16 case-control studies, with scores ranging from 5 to 9. Fourteen of these studies were classified as having a low risk of bias, while two were deemed to have a high risk of bias. Detailed information is available in Table 3, which outlines the Methodological Quality of Prospective Case-Control Studies according to the NOS criteria.

**Table 3:** The quality assessment of the included studies.

Included studies	Study population selection	Comparability between group	Comparability between group	Levels
Komlos et al. (2021)	✩✩✩✩	✩✩	✩✩✩	9✩
Chen et al. (2021)	✩✩✩	✩✩	✩✩✩	8✩
Shin et al. (2019)	✩✩✩	✩✩✩	✩✩	8✩
Laprise et al. (2016)	✩✩	✩✩	✩✩✩	7✩
Moraes et al. (2016)	✩✩	✩	✩✩	5✩
Narayan et al. (2014)	✩✩✩✩	✩✩	✩✩✩	9✩
Moergel et al. (2013)	✩✩✩✩	✩✩	✩✩	8✩
Tezal et al. (2009)	✩✩✩✩	✩✩	✩✩✩	9✩
De Rezende et al. (2008)	✩✩✩	✩✩✩	✩✩	8✩
Tezal et al. (2007)	✩✩✩✩	✩✩	✩✩✩	9✩
Chang et al. (2007)	✩✩✩✩	✩✩	✩✩✩	9✩
Guha et al. (2007)	✩✩✩	✩✩	✩✩✩	8✩
Rosenquist et al. (2005)	✩✩✩	✩	✩✩	6✩
Garrote et al. (2001)	✩✩✩✩	✩✩✩	✩✩	9✩
Bundgaard et al. (1995)	✩✩✩	✩✩	✩✩	7✩
Zheng et al. (1990)	✩✩✩✩	✩✩✩	✩✩	9✩

### Meta-analysis

#### The relationship between PD and OC

**Figure 2:**
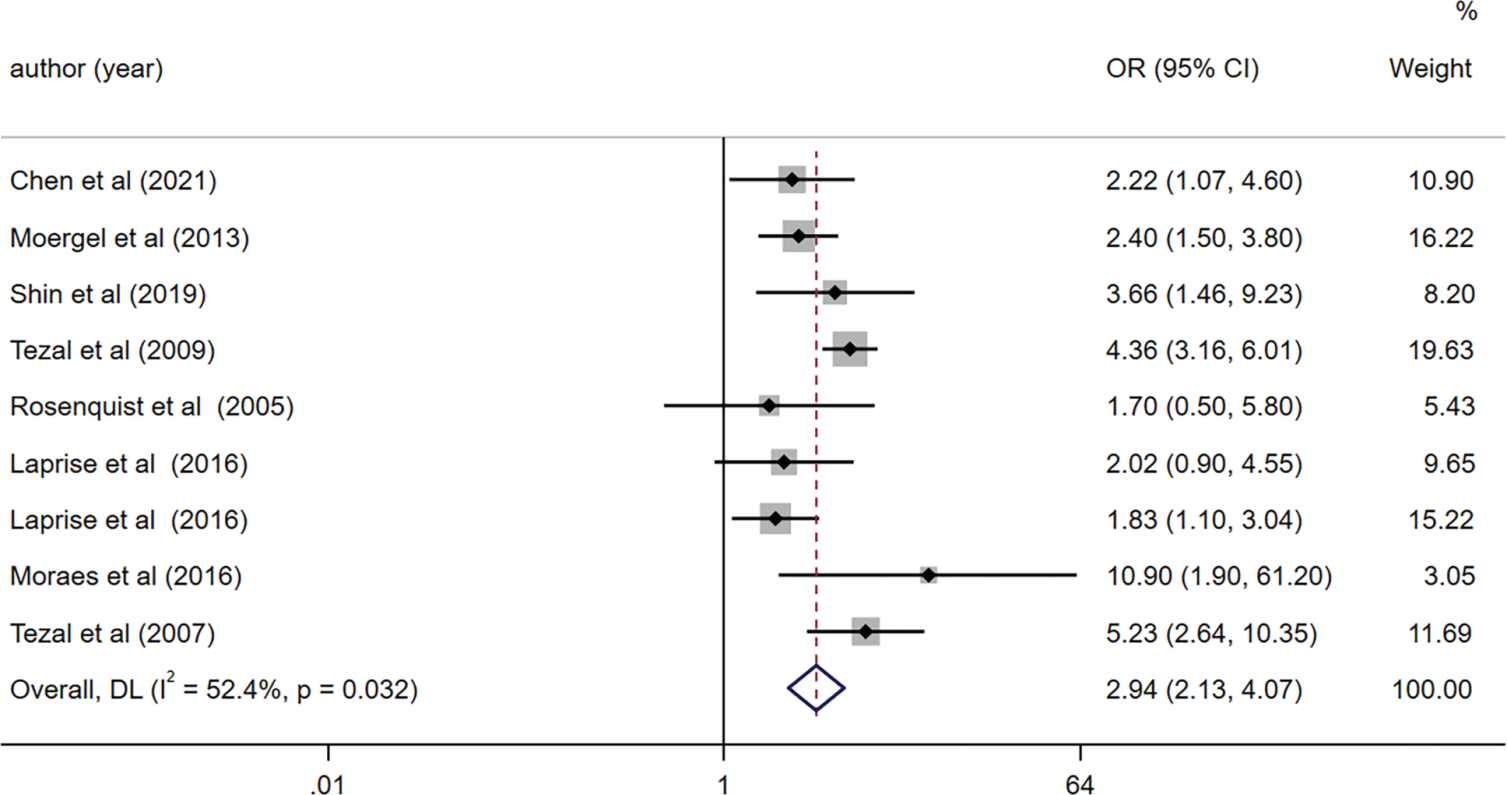
Forest plot of periodontal disease and risk of oral cancer (OC).

We incorporated a total of 16 studies into our qualitative analysis, all examining the relationship between PD and OC. However, for the meta-analysis, we pooled the OR from eight of these articles, specifically those that employed valid instruments to measure periodontitis [33, 34, 37, 39, 41, 45–47]. The pooled OR, generated via the random-effects model, is illustrated in Figure 2. The overall findings indicate a threefold rise in the likelihood of OC in populations afflicted with PD (OR = 2.94, 95% CI (2.13, 4.07), *p* < 0.001). Nevertheless, we detected variability among the studies included (*I*^2^ = 52.4%). No evidence of publication bias was observed (Egger’s test, *p* = 0.703), and the funnel plot displayed a symmetric distribution (Supplementary Figure S1).

We carried out sensitivity analyses by systematically excluding one study at a time.

These analyses consistently verified the reliability of the findings (OR = 2.63, 95% CI (1.92, 3.62), *p* < 0.001) even after excluding the study [34]. Heterogeneity across the remaining studies was negligible (*I*^2^ = 30.7%).

#### The relationship between missing teeth and OC

**Figure 3:**
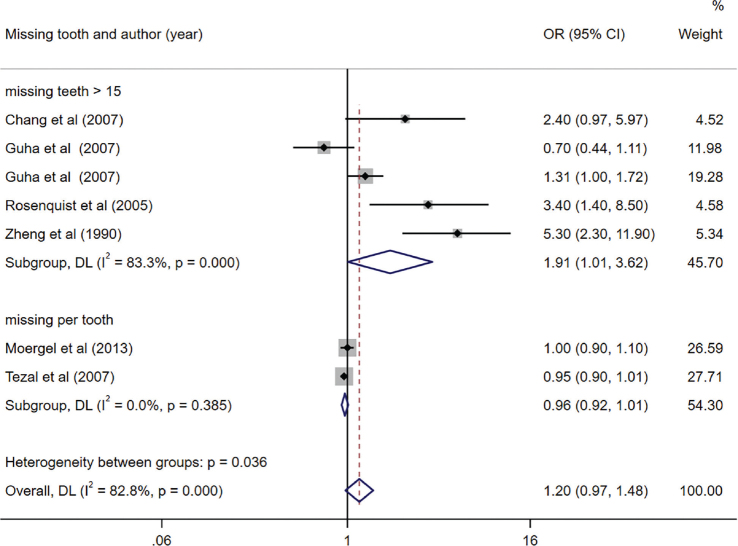
Forest plot of missing teeth and risk of oral cancer (OC).

Among the studies included in the meta-analysis, six specifically investigated the association between tooth loss and OC [35, 36, 41, 42, 45, 46]. We observed a significant link between having more than 15 missing teeth and an elevated risk of OC (OR = 1.91, 95% CI (1.01, 3.62), *p* = 0.047). However, no significant correlation was found between the number of missing teeth per tooth and the risk of OC (OR = 0.96, 95% CI (0.92, 1.01), *p* = 0.131). Notably, we identified significant heterogeneity among the studies investigating the impact of having more than 15 missing teeth (*I*^2^ = 83.3%). Notably, no indication of publication bias was observed in the included studies (Egger’s test, *p* = 0.054) (Figure 3).

#### Assessing the difference of PD between OC and control groups

**Figure 4:**
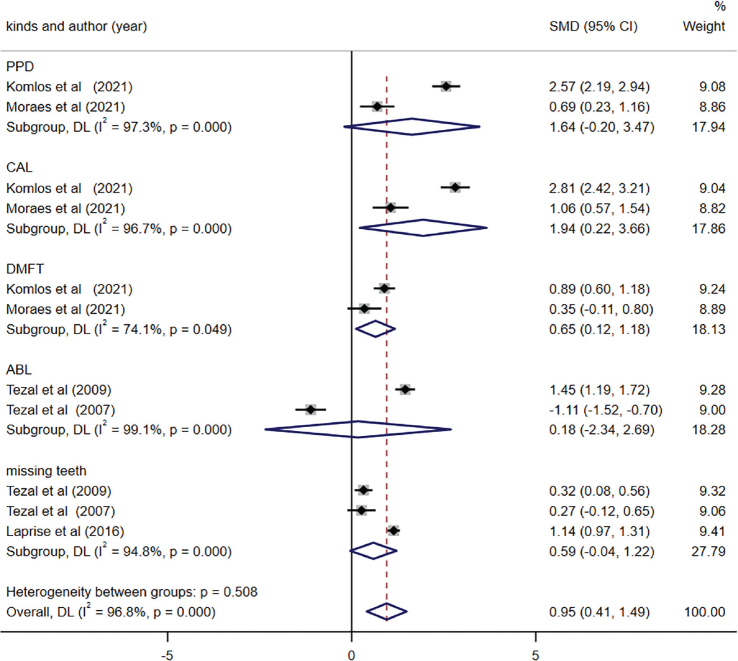
Forest plot of periodontal disease between oral cancer (OC) and control groups.

Among the included studies, only five studies evaluated the difference in PD difference between OC and control groups [33, 34, 37, 38, 45]. Depending on the various kinds of periodontal measurement parameters (i.e., PPD [periodontal pocket depth], ABL [alveolar bone lost], and missing teeth) reported in the included studies, subgroup analysis was conducted explicitly. The combined findings revealed a significant increase in CAL and DMFT (decayed, missing and filled teeth) index within OC group compared to the control group (CAL, SMD = 1.94, 95% CI (0.22, 3.66), *p* = 0.027; DMFT, SMD = 0.65, 95% CI (0.12, 1.18), *p* = 0.017). However, the remaining indicators of PD including PPD, ABL, and mean missing teeth, did not significantly differ between the two groups (Figure 4). Additionally, no evidence of publication bias was observed (egger, *p* = 0.837).

## Discussion

The current study was to assess the association between periodontitis and OC and the findings demonstrated that the risk of OC in patients with periodontitis increased 2.94 times compared to those without PD. Moreover, having more than 15 missing teeth emerged as a potential risk factor for OC, and there were significant differences that were observed in CAL and the DMFT index between OC patients and healthy controls.

Our findings have established a substantial association between the occurrence of OC and both periodontitis and tooth loss, aligning with the conclusions of the systematic review [50]. Since 2010, Seymour pointed out that inadequate oral health, particularly the degree and severity of PD, with numerous systemic diseases [16]. In 2012, Meisel and colleagues identified a potential connection between PD and premalignant oral lesions [51]. Recent evidence suggests that the degree and severity of PD and the presence of missing teeth may be linked to an increased risk of developing malignant diseases [52, 53]. Periodontitis is characterised as an inflammatory disease influencing the supporting structures of teeth, stands as the leading cause of tooth loss in adults. This chronic inflammatory condition holds the potential to elevate cancer risk by impeding apoptosis and promoting the growth of tumour cells [54]. The chronic inflammation induced by periodontal infections can also disrupt regular cell function and potentially contribute to carcinogenesis [55]. Consequently, periodontitis is considered as an indicator of a specific type of immune response that could impact the growth and advancement of cancer. Recent systematic reviews and meta-analyses exploring the relationship between PD and OC [25, 26, 50] consistently reveal a 2- to 5-fold elevated risk of developing OC among individuals with PD.

Previously, according to the inflammation of the disease, periodontitis was divided into mild, medium and severe forms. Severe periodontitis was often characterized by significant loss of periodontal support tissue, leading to loose or shifted teeth, and in severe cases, tooth dislodgment. In light of the epidemiology, aetiology, and pathogenesis of PD, as well as recent clinical evidence and related knowledge, the American Academy of Periodontology (AAP) and the European Federation of Periodontology (EFP) jointly organised the International Symposium on New Classifications of Periodontal and Periphytal Diseases in November 2017 [1]. As a result, a new classification system was formulated, and a consensus report was officially published in June 2018. Notably, the revised classification system eliminates the previous differentiation between chronic periodontitis and invasive periodontitis. Three distinct forms of periodontitis are now recognized: necrotizing periodontitis, periodontitis as a manifestation of systemic disease, and the forms of the disease previously acknowledged. A new periodontitis classification scheme is further characterised based on a multi-dimensional staging and grading system. Neither the previous classification nor the 2018 new classification changed the clinical definition of periodontitis: an inflammation that is microbial-related, host-mediated, and results in loss of periodontal adhesion. Most articles we retrieved did not use the 2018 new classification of periodontitis, but this did not affect the diagnosis of periodontitis. The aim is to classify the strongest scientific evidence, but in the absence of data, the use of lower-level evidence is inevitable.

We conducted a thorough search for publicly available studies pertaining to tooth loss and the risk of OC. Our investigation demonstrated that tooth loss indeed serves as a risk factor for OC, with an OR of 1.91. It is worth noting that tooth loss can be considered a potential indicator for PD, as the majority of adult tooth loss cases are attributed to periodontitis. However, the count of lost teeth can also be indicative of oral well-being and may arise from factors such as dental cavities, accidents, or orthodontic interventions. Consequently, tooth loss can encompass a range of oral health conditions, making it a somewhat imprecise indicator of periodontitis. Additionally, the prevalence of tooth loss can vary significantly among different population groups [52]. To date, several previous studies [35, 36, 41, 42, 46] have reported associations between tooth loss and the risk of OC; however, no meta-analysis has been conducted to investigate this connection. This might be due to the different categories of missing teeth. Some studies assessed the mean numbers of missing teeth (e.g., [41]), while Rosenquist et al. assessed the missing numbers of teeth over 20 and its risk of OC [46]. Meanwhile, another study used the standard of the number of missing teeth over 16 [42]. Additionally, certain studies examined the correlation between OC risk and the quantity of missing teeth per tooth [41, 45]. Future studies should use more standardised criteria to examine the connection between tooth loss and the risk of OC, and rigorously control for the underlying causes of patients’ missing teeth. Furthermore, the DMFT index is the key indicator of caries experience in dental epidemiology [56]. It represents the sum of decayed, missing (due to caries), and filled teeth in the permanent dentition. Dental caries results from demineralisation of teeth due to lactic acid produced by fermentation of carbohydrates by Gram-positive parthenogenic bacteria [57]. Previous research has highlighted the association between cariogenic bacteria and periodontal health, where reductions in the populations of these commensal bacteria are correlated with an increase in periodontal inflammation and destruction [58, 59], potentially contributing to OC. The meta-analysis results revealed significant differences in the DMFT index between OC patients and healthy controls. This finding aligns with previous studies suggesting an association between dental caries and head and neck cancers, particularly among patients with oral cavity and oropharyngeal cancers [60].

In addition, the findings also revealed a significant increase in CAL among OC patients in comparison to their healthy counterparts. Periodontitis is distinguished by the loss of periodontal ligament attachment, which is a consequence of host-mediated inflammation triggered by microbial activity. CAL, representing this loss of attachment, is determined by assessing the dentition using a standardised periodontal probe that measures from the cementoenamel junction to the base of the gingival sulcus [61]. However, both clinical and radiographic methods for CAL assessment and diagnosis have remained unaltered and are susceptible to inaccuracies, particularly in the initial phases of periodontitis [62]. Periodontal probing, utilised for CAL and probing depth assessment, is subject to various sources of variation, although relatively few articles have been published on this topic. Variability can arise from factors such as operator-related aspects (e.g., insertion force, probe placement, and angulation), probe design (which can affect tactile feedback), and the operator’s level of experience. All these elements contribute to the potential for uncertainty in periodontal measurements [63, 64]. As highlighted by Tezal and colleagues in 2005, the limitations of studies exploring PD as a risk factor for OC include the diversity of study designs, variations in study populations, and differences in measurement methods [65].

The present investigation, which explores the relationship between PD and the risk of OC, has several inherent limitations. The assessment of PD has proven to be a highly challenging task in previous research, mainly due to the necessity for multiple periodontal measurements and the evolving clinical definitions over time. The adoption of standardised clinical case definitions for population-based studies has been suggested [66, 67], although these definitions have not been widely employed in prior studies. Moreover, periodontitis is a multifaceted disease, and its diagnosis relies on determining CAL, probing depth, and bone loss. The diversity in measurement parameters has resulted in considerable heterogeneity among the studies included in this analysis. Additionally, the varying stages of periodontitis (e.g., mild, moderate, and severe periodontitis) were not assessed due to the limited data available in the incorporated studies. Future research should consider adopting standardized measurements and definitions to categorise and explore periodontitis along with its potential association with OC.

## Supplementary Material

Periodontitis and the risk of oral cancer: a meta-analysis of case-control studies

Periodontitis and the risk of oral cancer: a meta-analysis of case-control studies

## Data Availability

Data sharing not applicable to this article as no datasets were generated or analysed during the current study.
