# Supplementary material for: Periodontitis and the risk of oral cancer: a meta-analysis of case-control studies
Source: Acta Odontol Scand. 2024 May 14;83:40478. doi: 10.2340/aos.v83.40478 (PMC11302657; doi:10.2340/aos.v83.40478)
Supplement: Periodontitis and the risk of oral cancer: a meta-analysis of case-control studies [file AOS-83-40478-s1.pdf]

Supplementary material has been published as submitted. It has not been copyedited or typeset by Acta Odontologica Scandinavica.

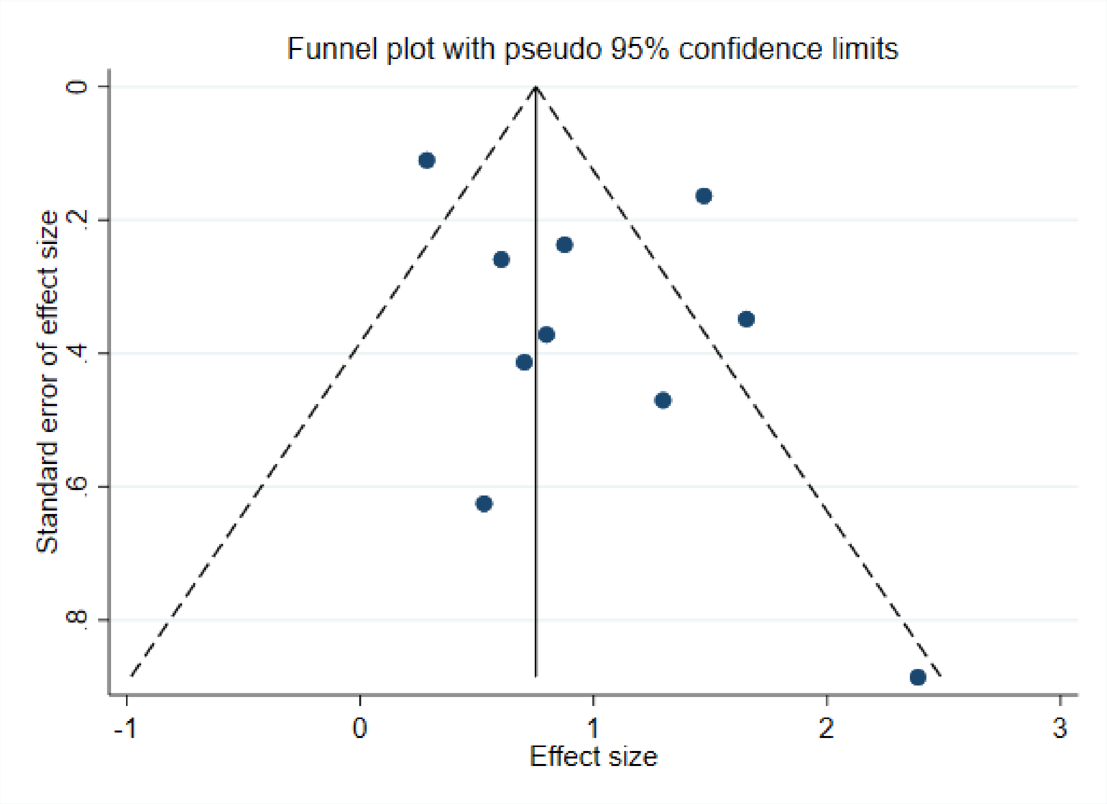

Figure S1. Funnel plot of the publication bias for the included studies assessing the association between periodontal disease and oral cancer.
