# Supplementary material for: Periodontitis and the risk of oral cancer: a meta-analysis of case-control studies
Source: Acta Odontol Scand. 2024 May 14;83:40478. doi: 10.2340/aos.v83.40478 (PMC11302657; doi:10.2340/aos.v83.40478)
Supplement: Periodontitis and the risk of oral cancer: a meta-analysis of case-control studies [file AOS-83-40478-s2.pdf]

Supplementary material has been published as submitted. It has not been copyedited or typeset by Acta Odontologica Scandinavica.

Table S1: Literature search strategy

1.Pubmed

Search number	Query	Records
#1	"Periodontitis"[Mesh]	34306
#2	(((((((((((((((((((((((((((((((((((((Periodontitis[Title/Abstract]) OR (Periodontitides[Title/Abstract])) OR (Pericementitis[Title/Abstract])) OR (Pericementitides[Title/Abstract])) OR (Paradontitis[Title/Abstract])) OR (Parodontitis[Title/Abstract])) OR (peridontitis[Title/Abstract])) OR (periodontal diseases[Title/Abstract])) OR (Disease, Periodontal[Title/Abstract])) OR (Diseases, Periodontal[Title/Abstract])) OR (Periodontal Disease[Title/Abstract])) OR (Parodontosis[Title/Abstract])) OR (Parodontoses[Title/Abstract])) OR (Pyorrhea Alveolaris[Title/Abstract])) OR (dental loss[Title/Abstract])) OR (dental migration[Title/Abstract])) OR (dental mobility[Title/Abstract])) OR (furcation defects[Title/Abstract])) OR (mesial movement of teeth[Title/Abstract])) OR (paradontal disease[Title/Abstract])) OR (paradontopathy[Title/Abstract])) OR (paraodontopathy[Title/Abstract])) OR (parodentopathy[Title/Abstract])) OR (parodontal disease[Title/Abstract])) OR (parodontium disease[Title/Abstract])) OR (parodontive tissue disease[Title/Abstract])) OR (peridontal disease[Title/Abstract])) OR (peridontal tissue disease[Title/Abstract])) OR (peridontium disease[Title/Abstract])) OR (periodontal atrophy[Title/Abstract])) OR (periodontal attachment loss[Title/Abstract])) OR (periodontal infection[Title/Abstract])) OR (periodontium disease[Title/Abstract])) OR (periodontopathy[Title/Abstract])) OR (tooth loss[Title/Abstract])) OR (tooth migration[Title/Abstract])) OR (tooth mobility'[Title/Abstract])) OR (tooth movement[Title/Abstract])	70428
#3	"Mouth Neoplasms"[Mesh]	76064
#4	((Mouth Neoplasms[Title/Abstract]) OR (Mouth Neoplasm[Title/Abstract])) OR (Neoplasm, Mouth[Title/Abstract])) OR (Neoplasms, Oral[Title/Abstract])) OR (Neoplasm, Oral[Title/Abstract])) OR (Oral Neoplasm[Title/Abstract])) OR (Oral Neoplasms[Title/Abstract])) OR (Neoplasms, Mouth[Title/Abstract])) OR (Cancer of Mouth[Title/Abstract])) OR (Mouth Cancers[Title/Abstract])) OR (Oral Cancer[Title/Abstract])) OR (Cancer, Oral[Title/Abstract])) OR (Cancers, Oral[Title/Abstract])) OR (Oral Cancers[Title/Abstract])) OR (Cancer of the Mouth[Title/Abstract])) OR (Mouth Cancer[Title/Abstract])) OR (Cancer, Mouth[Title/Abstract])) OR (Cancers, Mouth[Title/Abstract])) OR (cancer, mouth[Title/Abstract])) OR (intraoral cancer[Title/Abstract])) OR (mouth mucosa cancer[Title/Abstract])) OR (oral cancer[Title/Abstract])) OR (oral cavity cancer[Title/Abstract])) OR (mouth tumor[Title/Abstract])) OR (buccal mucosa tumor[Title/Abstract])) OR (buccal mucosa tumour[Title/Abstract])) OR (intraoral tumor[Title/Abstract])) OR (intraoral tumour[Title/Abstract])) OR (mouth cavity tumor[Title/Abstract])) OR (mouth cavity	29812

	tumour[Title/Abstract])) OR (mouth neoplasm[Title/Abstract])) OR (mouth neoplasms[Title/Abstract])) OR (mouth tumour[Title/Abstract])) OR (oral cavity tumor[Title/Abstract])) OR (oral cavity tumour[Title/Abstract])) OR (oral mucosa tumor[Title/Abstract])) OR (oral mucosa tumour[Title/Abstract])) OR (oral tumor[Title/Abstract])) OR (oral tumour[Title/Abstract])) OR (tumor, mouth[Title/Abstract])) OR (tumour, mouth[Title/Abstract])	
#5	("Periodontitis"[Mesh]) OR ((((((((((((((((((((((((((((((((((((((Periodontitis[Title/Abstract]) OR (Periodontitides[Title/Abstract])) OR (Pericementitis[Title/Abstract])) OR (Pericementitides[Title/Abstract])) OR (Paradontitis[Title/Abstract])) OR (Parodontitis[Title/Abstract])) OR (peridontitis[Title/Abstract])) OR (periodontal diseases[Title/Abstract])) OR (Disease, Periodontal[Title/Abstract])) OR (Diseases, Periodontal[Title/Abstract])) OR (Periodontal Disease[Title/Abstract])) OR (Parodontosis[Title/Abstract])) OR (Parodontoses[Title/Abstract])) OR (Pyorrhea Alveolaris[Title/Abstract])) OR (dental loss[Title/Abstract])) OR (dental migration[Title/Abstract])) OR (dental mobility[Title/Abstract])) OR (furcation defects[Title/Abstract])) OR (mesial movement of teeth[Title/Abstract])) OR (paradontal disease[Title/Abstract])) OR (paradontopathy[Title/Abstract])) OR (paraodontopathy[Title/Abstract])) OR (parodentopathy[Title/Abstract])) OR (parodontal disease[Title/Abstract])) OR (parodontium disease[Title/Abstract])) OR (parodontive tissue disease[Title/Abstract])) OR (peridontal disease[Title/Abstract])) OR (peridontal tissue disease[Title/Abstract])) OR (peridontium disease[Title/Abstract])) OR (periodontal atrophy[Title/Abstract])) OR (periodontal attachment loss[Title/Abstract])) OR (periodontal infection[Title/Abstract])) OR (periodontium disease[Title/Abstract])) OR (periodontopathy[Title/Abstract])) OR (tooth loss[Title/Abstract])) OR (tooth migration[Title/Abstract])) OR (tooth mobility'[Title/Abstract])) OR (tooth movement[Title/Abstract]))	81070
#6	("Mouth Neoplasms"[Mesh]) OR (((Mouth Neoplasms[Title/Abstract]) OR (Mouth Neoplasm[Title/Abstract])) OR (Neoplasm, Mouth[Title/Abstract])) OR (Neoplasms, Oral[Title/Abstract])) OR (Neoplasm, Oral[Title/Abstract])) OR (Oral Neoplasm[Title/Abstract])) OR (Oral Neoplasms[Title/Abstract])) OR (Neoplasms, Mouth[Title/Abstract])) OR (Cancer of Mouth[Title/Abstract])) OR (Mouth Cancers[Title/Abstract])) OR (Oral Cancer[Title/Abstract])) OR (Cancer, Oral[Title/Abstract])) OR (Cancers, Oral[Title/Abstract])) OR (Oral Cancers[Title/Abstract])) OR (Cancer of the Mouth[Title/Abstract])) OR (Mouth Cancer[Title/Abstract])) OR (Cancer, Mouth[Title/Abstract])) OR (Cancers, Mouth[Title/Abstract])) OR (cancer, mouth[Title/Abstract])) OR (intraoral cancer[Title/Abstract])) OR (mouth mucosa cancer[Title/Abstract])) OR (oral cancer[Title/Abstract])) OR (oral cavity cancer[Title/Abstract])) OR (mouth tumor[Title/Abstract])) OR (buccal mucosa tumor[Title/Abstract])) OR (buccal mucosa tumour[Title/Abstract])) OR (intraoral tumor[Title/Abstract])) OR (intraoral tumour[Title/Abstract])) OR (mouth cavity tumor[Title/Abstract])) OR (mouth cavity tumour[Title/Abstract])) OR (mouth neoplasm[Title/Abstract])) OR (mouth neoplasms[Title/Abstract])) OR (mouth tumour[Title/Abstract])) OR (oral cavity tumor[Title/Abstract])) OR (oral cavity	88554

	tumour[Title/Abstract])) OR (oral mucosa tumor[Title/Abstract])) OR (oral mucosa tumour[Title/Abstract])) OR (oral tumor[Title/Abstract])) OR (oral tumour[Title/Abstract])) OR (tumor, mouth[Title/Abstract])) OR (tumour, mouth[Title/Abstract]))	
#7	(("Periodontitis"[Mesh]) OR (((((((((((((((((((((((((((((((((((((((Periodontitis[Title/Abstract]) OR (Periodontitides[Title/Abstract])) OR (Pericementitis[Title/Abstract])) OR (Pericementitides[Title/Abstract])) OR (Paradontitis[Title/Abstract])) OR (Parodontitis[Title/Abstract])) OR (peridontitis[Title/Abstract])) OR (periodontal diseases[Title/Abstract])) OR (Disease, Periodontal[Title/Abstract])) OR (Diseases, Periodontal[Title/Abstract])) OR (Periodontal Disease[Title/Abstract])) OR (Parodontosis[Title/Abstract])) OR (Parodontoses[Title/Abstract])) OR (Pyorrhea Alveolaris[Title/Abstract])) OR (dental loss[Title/Abstract])) OR (dental migration[Title/Abstract])) OR (dental mobility[Title/Abstract])) OR (furcation defects[Title/Abstract])) OR (mesial movement of teeth[Title/Abstract])) OR (paradontal disease[Title/Abstract])) OR (paradontopathy[Title/Abstract])) OR (paraodontopathy[Title/Abstract])) OR (parodentopathy[Title/Abstract])) OR (parodontal disease[Title/Abstract])) OR (parodontium disease[Title/Abstract])) OR (parodontive tissue disease[Title/Abstract])) OR (peridontal disease[Title/Abstract])) OR (peridontal tissue disease[Title/Abstract])) OR (peridontium disease[Title/Abstract])) OR (periodontal atrophy[Title/Abstract])) OR (periodontal attachment loss[Title/Abstract])) OR (periodontal infection[Title/Abstract])) OR (periodontium disease[Title/Abstract])) OR (periodontopathy[Title/Abstract])) OR (tooth loss[Title/Abstract])) OR (tooth migration[Title/Abstract])) OR (tooth mobility'[Title/Abstract])) OR (tooth movement[Title/Abstract])))) AND (("Mouth Neoplasms"[Mesh]) OR ((Mouth Neoplasms[Title/Abstract]) OR (Mouth Neoplasm[Title/Abstract])) OR (Neoplasm, Mouth[Title/Abstract])) OR (Neoplasms, Oral[Title/Abstract])) OR (Neoplasm, Oral[Title/Abstract])) OR (Oral Neoplasm[Title/Abstract])) OR (Oral Neoplasms[Title/Abstract])) OR (Neoplasms, Mouth[Title/Abstract])) OR (Cancer of Mouth[Title/Abstract])) OR (Mouth Cancers[Title/Abstract])) OR (Oral Cancer[Title/Abstract])) OR (Cancer, Oral[Title/Abstract])) OR (Cancers, Oral[Title/Abstract])) OR (Oral Cancers[Title/Abstract])) OR (Cancer of the Mouth[Title/Abstract])) OR (Mouth Cancer[Title/Abstract])) OR (Cancer, Mouth[Title/Abstract])) OR (Cancers, Mouth[Title/Abstract])) OR (cancer, mouth[Title/Abstract])) OR (intraoral cancer[Title/Abstract])) OR (mouth mucosa cancer[Title/Abstract])) OR (oral cancer[Title/Abstract])) OR (oral cavity cancer[Title/Abstract])) OR (mouth tumor[Title/Abstract])) OR (buccal mucosa tumor[Title/Abstract])) OR (buccal mucosa tumour[Title/Abstract])) OR (intraoral tumor[Title/Abstract])) OR (intraoral tumour[Title/Abstract])) OR (mouth cavity tumor[Title/Abstract])) OR (mouth cavity tumour[Title/Abstract])) OR (mouth neoplasm[Title/Abstract])) OR (mouth neoplasms[Title/Abstract])) OR (mouth tumour[Title/Abstract])) OR (oral cavity tumor[Title/Abstract])) OR (oral cavity tumour[Title/Abstract])) OR (oral mucosa tumor[Title/Abstract])) OR (oral mucosa tumour[Title/Abstract])) OR (oral	1065

	tumor[Title/Abstract])) OR (oral tumour[Title/Abstract])) OR (tumor, mouth[Title/Abstract])) OR (tumour, mouth[Title/Abstract])))

2.Cochrane

Search number	Query
#1	MeSH descriptor: [Periodontitis] explode all trees
#2	(Periodontitis):ti,ab,kw OR (Periodontitides):ti,ab,kw OR (Pericementitis):ti,ab,kw OR (Pericementitides):ti,ab,kw OR (Paradontitis):ti,ab,kw
#3	(Parodontitis):ti,ab,kw OR (peridontitis):ti,ab,kw OR (periodontal diseases):ti,ab,kw OR (Disease, Periodontal):ti,ab,kw OR (Diseases, Periodontal):ti,ab,kw
#4	(Periodontal Disease):ti,ab,kw OR (Parodontosis):ti,ab,kw OR (Parodontoses):ti,ab,kw OR (Pyorrhea Alveolaris):ti,ab,kw OR (dental loss):ti,ab,kw
#5	(dental migration):ti,ab,kw OR (dental mobility):ti,ab,kw OR (furcation defects):ti,ab,kw OR (mesial movement of teeth):ti,ab,kw OR (paradontal disease):ti,ab,kw
#6	(paradontopathy):ti,ab,kw OR (paraodontopathy'):ti,ab,kw OR (parodentopathy):ti,ab,kw OR (parodontal disease):ti,ab,kw OR (parodontium disease):ti,ab,kw
#7	(parodontive tissue disease):ti,ab,kw OR (peridontal disease):ti,ab,kw OR (peridontal tissue disease):ti,ab,kw OR (peridontium disease):ti,ab,kw OR (periodontal atrophy):ti,ab,kw
#8	(periodontal attachment loss):ti,ab,kw OR (periodontal infection'):ti,ab,kw OR (periodontium disease):ti,ab,kw OR (periodontopathy):ti,ab,kw OR (tooth loss'):ti,ab,kw
#9	(tooth migration):ti,ab,kw OR (tooth mobility):ti,ab,kw OR (tooth movement):ti,ab,kw
#10	#1 or #2 or #3 or #4 or #5 or #6 or #7 or #8 or #9
#11	MeSH descriptor: [Mouth Neoplasms] explode all trees
#12	(Mouth Neoplasms):ti,ab,kw OR (Mouth Neoplasm):ti,ab,kw OR (Neoplasm, Mouth):ti,ab,kw OR (Neoplasms, Oral):ti,ab,kw OR (Neoplasm, Oral):ti,ab,kw
#13	(Oral Neoplasm):ti,ab,kw OR (Oral Neoplasms):ti,ab,kw OR (Neoplasms, Mouth):ti,ab,kw OR (Cancer of Mouth):ti,ab,kw OR (Mouth Cancers):ti,ab,kw
#14	(Oral Cancer):ti,ab,kw OR (Cancer, Oral):ti,ab,kw OR (Cancers, Oral):ti,ab,kw OR (Oral Cancers):ti,ab,kw OR (Cancer of the Mouth):ti,ab,kw
#15	(Mouth Cancer):ti,ab,kw OR (Cancer, Mouth):ti,ab,kw OR (Cancers, Mouth):ti,ab,kw OR (cancer, mouth):ti,ab,kw OR (intraoral cancer):ti,ab,kw
#16	(mouth mucosa cancer):ti,ab,kw OR (oral cancer):ti,ab,kw OR (oral cavity cancer):ti,ab,kw OR (mouth tumor):ti,ab,kw OR (buccal mucosa tumor):ti,ab,kw
#17	(buccal mucosa tumour):ti,ab,kw OR (intraoral tumor):ti,ab,kw OR (intraoral tumour):ti,ab,kw OR (mouth cavity tumor):ti,ab,kw OR (mouth cavity tumour):ti,ab,kw
#18	(mouth neoplasm):ti,ab,kw OR (mouth neoplasms):ti,ab,kw OR (mouth tumour):ti,ab,kw OR (oral cavity tumor):ti,ab,kw OR (oral cavity tumour):ti,ab,kw
#19	(oral mucosa tumor):ti,ab,kw OR (oral mucosa tumour):ti,ab,kw OR (oral tumor):ti,ab,kw OR (oral tumour):ti,ab,kw OR (tumor, mouth):ti,ab,kw
#20	(tumour, mouth):ti,ab,kw
#21	#11 or #12 or #13 or #14 or #15 or #16 or #17 or #18 or #19 or #20
#22	#10 and #21

3.Embase

Search number	Query	Records
#1	'periodontitis'/exp	53364
#2	'periodontitis'/exp OR periodontitis OR periodontitides:ab,ti OR pericementitis:ab,ti OR pericementitides:ab,ti OR paradontitis:ab,ti OR parodontitis:ab,ti OR peridontitis:ab,ti OR 'periodontal diseases':ab,ti OR 'disease, periodontal':ab,ti OR 'diseases, periodontal':ab,ti OR 'periodontal disease':ab,ti OR parodontosis:ab,ti OR parodontoses:ab,ti OR 'pyorrhea alveolaris':ab,ti OR 'dental loss':ab,ti OR 'dental migration':ab,ti OR 'dental mobility':ab,ti OR 'furcation defects':ab,ti OR 'mesial movement of teeth':ab,ti OR 'paradontal disease':ab,ti OR paradontopathy:ab,ti OR paraodontopathy:ab,ti OR parodentopathy:ab,ti OR 'parodontal disease':ab,ti OR 'parodontium disease':ab,ti OR 'parodontive tissue disease':ab,ti OR 'peridontal disease':ab,ti OR 'peridontal tissue disease':ab,ti OR 'peridontium disease':ab,ti OR 'periodontal atrophy':ab,ti OR 'periodontal attachment loss':ab,ti OR 'periodontal infection':ab,ti OR 'periodontium disease':ab,ti OR periodontopathy:ab,ti OR 'tooth loss':ab,ti OR 'tooth migration':ab,ti OR 'tooth mobility':ab,ti OR 'tooth movement':ab,ti	85492
#3	#1 OR #2	85492
#4	'mouth tumor'/exp	148941
#5	'mouth neoplasms'/exp OR 'mouth neoplasms' OR (('mouth'/exp OR mouth) AND ('neoplasms'/exp OR neoplasms)) OR 'neoplasm, mouth':ab,ti OR 'neoplasms, oral':ab,ti OR 'neoplasm, oral':ab,ti OR 'oral neoplasm':ab,ti OR 'oral neoplasms':ab,ti OR 'neoplasms, mouth':ab,ti OR 'cancer of mouth':ab,ti OR 'mouth cancers':ab,ti OR 'cancer, oral':ab,ti OR 'cancers, oral':ab,ti OR 'oral cancers':ab,ti OR 'cancer of the mouth':ab,ti OR 'cancers, mouth':ab,ti OR 'mouth cancer':ab,ti OR 'cancer, mouth':ab,ti OR 'intraoral cancer':ab,ti OR 'mouth mucosa cancer':ab,ti OR 'oral cancer':ab,ti OR 'oral cavity cancer':ab,ti OR 'mouth tumor':ab,ti OR 'buccal mucosa tumor':ab,ti OR 'buccal mucosa tumour':ab,ti OR 'intraoral tumor':ab,ti OR 'intraoral tumour':ab,ti OR 'mouth cavity tumor':ab,ti OR 'mouth cavity tumour':ab,ti OR 'mouth neoplasm':ab,ti OR 'mouth neoplasms':ab,ti OR 'mouth tumour':ab,ti OR 'oral cavity tumor':ab,ti OR 'oral cavity tumour':ab,ti OR 'oral mucosa tumor':ab,ti OR 'oral mucosa tumour':ab,ti OR 'oral tumor':ab,ti OR 'oral tumour':ab,ti OR 'tumor, mouth':ab,ti OR 'tumour, mouth':ab,ti	209985
#6	#4 OR #5	209985
#7	#3 AND #6	2636

4.Web of science

Search number	Query
#1	**Periodontitis** (MeSH) or **Periodontitides** (MeSH) or **Pericementitis** (MeSH) or **Pericementitides** (MeSH) or **Paradontitis** (MeSH) or **Parodontitis** (MeSH) or **peridontitis** (MeSH) or **periodontal diseases** (MeSH) or **Disease, Periodontal** (MeSH) or **Diseases, Periodontal** (MeSH) or **Periodontal Disease** (MeSH) or **Parodontosis** (MeSH) or **Parodontoses** (MeSH) or **Pyorrhea Alveolaris** (MeSH) or **dental loss** (MeSH) or **dental migration** (MeSH) or **dental mobility** (MeSH) or **furcation defects** (MeSH) or **mesial movement of teeth** (MeSH) or **paradontal disease** (MeSH) or **paradontopathy** (MeSH) or **paraodontopathy** (MeSH) or **parodentopathy** (MeSH

	) or **parodontal disease** (MeSH) or **parodontium disease** (MeSH) or **parodontive tissue disease** (MeSH) or **peridontal disease** (MeSH) or **peridontal tissue disease** (MeSH) or **peridontium disease** (MeSH) or **periodontal atrophy** (MeSH) or **periodontal attachment loss** (MeSH) or **periodontal infection** (MeSH) or **periodontium disease** (MeSH) or **periodontopathy** (MeSH) or **tooth loss** (MeSH) or **tooth migration** (MeSH) or **tooth mobility** (MeSH) or **tooth movement** (MeSH)
**#2**	Mouth Neoplasms **(MeSH) or** Mouth Neoplasm **(MeSH) or** Neoplasm, Mouth **(MeSH) or** Neoplasms, Oral **(MeSH) or** Neoplasm, Oral **(MeSH) or** Oral Neoplasm **(MeSH) or** Oral Neoplasms **(MeSH) or** Neoplasms, Mouth **(MeSH) or** Cancer of Mouth **(MeSH) or** Mouth Cancers **(MeSH) or** Oral Cancer **(MeSH) or** Cancer, Oral **(MeSH) or** Cancers, Oral **(MeSH) or** Oral Cancers **(MeSH) or** Cancer of the Mouth **(MeSH) or** Mouth Cancer **(MeSH) or** Cancer, Mouth **(MeSH) or** Cancers, Mouth **(MeSH) or** cancer, mouth **(MeSH) or** intraoral cancer **(MeSH) or** mouth mucosa cancer **(MeSH) or** oral cancer **(MeSH) or** oral cavity cancer **(MeSH) or** mouth tumor **(MeSH) or** buccal mucosa tumor **(MeSH) or** buccal mucosa tumour **(MeSH) or** intraoral tumor **(MeSH) or** intraoral tumour **(MeSH) or** mouth cavity tumor **(MeSH) or** mouth cavity tumour **(MeSH) or** mouth neoplasm **(MeSH) or** mouth neoplasms **(MeSH) or** mouth tumour **(MeSH) or** oral cavity tumor **(MeSH) or** oral cavity tumour **(MeSH) or** oral mucosa tumor **(MeSH) or** oral mucosa tumour **(MeSH) or** oral tumor **(MeSH) or** oral tumour **(MeSH) or** tumor, mouth **(MeSH) or** tumour, mouth **(MeSH)**
#3	#1 AND #2
